# The Overlapping Roles of Antimicrobial Peptides and Complement in Recruitment and Activation of Tumor-Associated Inflammatory Cells

**DOI:** 10.3389/fimmu.2015.00002

**Published:** 2015-01-22

**Authors:** Izzat A. M. Al-Rayahi, Raghad H. H. Sanyi

**Affiliations:** ^1^Department of Infection, Immunity and Inflammation, College of Medicine, Biological Sciences and Psychology, University of Leicester, Leicester, UK; ^2^The Ministry of Higher Education, Baghdad, Iraq

**Keywords:** defensins, cathelicidins, anti-tumor activity, c5a, cell recruitment and activation

## Abstract

Antimicrobial peptides (AMPs) represent a group of small (6–100 amino acids), biologically active molecules, which are produced by plants, mammals, and microorganisms (1). An important element of the innate immune response, AMP, possesses potent antibiotic, antifungal, and antiviral activities. Furthermore, AMP may be involved in a number of other processes such as angiogenesis and modulation of the immune response such as stimulation of chemokines and chemotaxis of leukocytes. AMPs have been proposed as alternative therapies for infectious diseases. AMP may also exert cytotoxic activity against tumor cells. Further understanding of the biological function of these peptides during tumor development and progression may aid in the development of novel anti-tumor therapies with refined application of innate molecules. AMP and complement have distinct roles to play in shaping the microenvironment (Table 1). Components of the complement system are integral contributors in responding to infection and sterile inflammation. Moreover, complement plays a role in the trafficking of cells in the tumor microenvironment, and thereby possibly in the immune response to cancer. This article will try to outline characteristics of AMP and complement in mobilization and recruitment of cells in tumor microenvironment.

## Defensins, an Example of Antimicrobial Peptides with a Controversial Role in Cancer

The defensin family includes three subfamilies (α, β, and θ defensins). Defensins can be stored in cytoplasmic granules of neutrophils and in macrophages or can be released into the extracellular environment from epithelial cells and mesothelial cells. In humans, there are 6 α defensins and 11 β defensins, exhibiting a wide range of antimicrobial, but also additional biological activities: β defensins possess a proinflammatory effect via binding a variety of receptors. For example, human β defensin 2 (hBD-2) and, to a lesser extent, human β defensin 1 (hBD1) bind to CCR6 resulting in increased chemoattraction of both CD4^+^ memory T cells and immature dendritic cells (2). β defensins can also play a role in tumorigenesis. Altered expression of β defensins was seen in epithelial cell-derived cancers such as prostatic cancer, basal cell carcinoma, oral squamous cell carcinoma (OSCC) and renal cell carcinoma. Expression of hBD1 was found to be diminished or suppressed in malignant prostate tissue compared to high or moderate expression of hBD1 in surrounding benign tissue (3). This altered expression of β defensins led researchers to investigate anti-tumor activity of β defensins. Results showed that induction of hBD1 expression in prostatic cancer cell line lead to increased cell death inferring that hBD1 is anti-tumorigenic and that loss or decrease in expression may lead to tumor progression. In this sense, it was found that diminished expression of hBD1 was found associated with worse grading of prostate cancer (4).

**Table 1:** **Tumors develop a complex network of cellular interactions involving both proinflammatory and suppressive cells**.

Immunosuppressive	Proinflammatory
C5a/C5aR → MDSC recruitment	C5a attract CD8^+^ T-cells
AMP involved in angiogenesis	AMP exert cytotoxic activity
↓Expression of human β defensin 1 → tumor progression (prostate cancer)	↑ hBD3 associate with recruitment TAM (lung cancer, renal cell carcinoma, and OSCC)
HPN1 inhibition Classical and Lectin pathway	β defensins bind to a variety of receptors (CCR2)
Express α defensins (HPN1-3) in endothelial → angiogenesis	hBD1 bind to CCR6 resulting in ↑ CD4^+^
Express α defensins (HPN1-3) in endothelial → angiogenesis	Blockage of C5aR ↓ decrease tumor growth
Deficiency of LL37aid in inflammation and tumor progression	Overexpression hBD3 → chemoattract monocytes tumor microenvironment
	↑ ↑ ↑ HPN1-3 → cytotoxic effect on cancer cell line

However, the role of defensins in tumor development and tumor progression has been controversial with some groups reporting overexpression of some β defensins in some types of cancer. High levels of hBD1 and hBD-2 were found in sera of patients with lung cancer. Another example is the overexpression of hBD1 in renal cell carcinoma and hBD3 in OSCC (5, 6).

Further investigation into the role of hBD3 in mediating the tumor-related inflammatory process in oral cancer revealed that overexpression of hBD3 was associated with recruitment and accumulation of tumor-associated macrophages (TAM). These cells represent a significant part of infiltrating immune cells in the tumor microenvironment and are thought to play a role in the development and progression of tumors, but are probably a heterogeneous population (7). Moreover, hBD3 overexpression stimulated the expression of interleukin-1α (IL-1α), IL-6, IL-8, CCL18, and tumor necrosis factor-α (TNF-α). HBD3 chemoattract monocytes to the tumor microenvironment via chemokine receptor 2 (CCR2), further supporting the role of human β defensins in establishing the tumor microenvironment, which leads to tumor progression (8). By increasing vascularization, HBD increase tumor angiogenesis in SCC microenvironment (9). Conejo-Garcia and coworkers described a mechanism in which β defensins support tumor vasculogenesis through recruitment of dendritic cell precursors. Tumor vascularization and growth were enhanced in the presence of increased Vegf-A expression (10).

It was also found that HBD3 has the ability to inhibit the migration of colon cancer cells in a dose-dependent manner, which means that HBD3 could be a potential new drug for treatment of colon cancer (11).

Other reported activities of defensins include activation of the classical pathway of complement. The interaction with the complement system occurs via both C1q-dependent and C1q-independent mechanisms (12, 13). In contrast, other studies have reported that HBD-2, which has a structural homology with a C1q inhibitory molecule, can significantly inhibit the classical pathway of complement (14). Some defensins may inhibit both the classical and lectin pathways of complement. Human neutrophilic peptide 1 (HPN1) was found to bind to C1q and MBL leading to inhibition of these two complement activation pathways (15).

Human β defensins have not been the only group of defensins associated with tumor tissue. In the last few years, α defensins were detected in malignant tissue and fluids (serum and plasma) from patients with cancer. Human neutrophilic peptides 1, 2, and 3 (HNP 1–3), members of α defensins antimicrobial peptides (AMP), are expressed in endothelial cells in several tumor types suggesting that angiogenesis could be effected by α-defensins (16). The role of α defensins in cancer has also been controversial with some *in vitro* studies suggesting that supranormal (>25 μg/ml) levels of HNP1–3 have a cytotoxic effect on cancer cell lines and physiologic levels (6–25 μg/ml) of HNP 1–3 increase the proliferation and invasiveness of cancer cell lines (17), while others show that α defensin expression associates with greater invasiveness *in vivo* and *in vitro* (18).

## Complement and Cancer

The complement system is an important element of the innate immunity with a well-established role in acute inflammation and continuous activation in chronic inflammation. In addition, components of the complement system mediate cellular regeneration and growth (bone and cartilage development, neurogenesis, bone marrow engraftment and regeneration of the liver) (19, 20). The complement activated system play a dual role in the tumor microenvironment. Traditionally, it was assumed that components of the complement system play a role in anti-tumor immune response either through complement mediated cytotoxicity or via antibody-dependent cell-mediated cytotoxicity. However, work has suggested that components of the complement system may also cause immune suppression and enhance tumor growth which means that the role of complement components in tumor growth should be revisited. Complement anaphylatoxins C3a and C5a play a complex role in tumor growth by inhibit antigen-specific CD8 + T cell-mediated anti-tumor immune responses. In their study of a murine TC-1 syngeneic model of cervical cancer, blockage of C5aR resulted in decreased tumor growth. Coinciding with an increase in infiltrating CD8^+^ cytotoxic T cells and inhibition of MDSC recruitment (21), the effect of C5aR blockade was similar to that seen after treatment with a well-known anti-tumor chemotherapeutic agent. Moreover, a similar degree of inhibition in tumor growth was also noted in C5aR-deficient mice. MDSCs isolated from C5aR-deficient tumor bearing mice lacked the ability to inhibit CD3^+^ T cells proliferation seen in C5aR wild-type mice (21). Overactivation of the infiltrating cells due to high concentration of C5a may result in suppression of anti-tumor T cells, thus leading to progression of tumors. On the other hand, low concentration of C5a can lead to a powerful anti-tumor immune response (22).

These findings present complement inhibition as an option for developing novel anti-cancer therapies, which lacks the side effects of the conventional anti-cancer therapies. The concomitant inhibition of egress of cells from the bone marrow by interfering with C5a/C5aR interactions, however, carries a possible risk of compromised inflammatory response to infection.

C3 deficiency leads to altered immune response in experimental ovarian cancer causing a decrease in tumor development and progression (23).

## Cathelicidins, a Group of Antimicrobial Peptides with Miscellaneous Biological Functions

Cathelicidins constitute a mammalian antimicrobial peptide family. Cathelicidins are characterized by a variable antimicrobial peptide on the C-terminus and a moderately conserved N-terminal cathelin domain. The cationic property of cathelicidin enables it to electrostatically react with the anionic membrane of microbes and some tumor cells resulting in disruption of cell membranes, which eventually leads to cell death. This process results in destruction of tumor cells (and microbes), while normal cells are left intact. In humans, the only known cathelicidin is the hCAP-18/LL37. After stimulation, hCAP-18 is cleaved producing a peptide containing 37 amino acids starting with two leucines termed LL37. Expression of the hCAP-18 gene was found in squamous epithelium of intestine, mouth, cervix, tongue, esophagus, and the airways. In addition, LL37 is produced by immature neutrophils, natural killer cells, B cells, monocytes, and mast cells. LL37 plays a protective role in preventing bacterial inflammations. This conclusion came from the observation that LL37 is down regulated in patients infected with *Shigella*. A number of studies have pointed out that cathelicidin could play an important role in preventing bacteria-related inflammation and perhaps also carcinogenesis. An example of this is infection of the gut with *Helicobacter pylori*, where LL37 is overexpressed in the early stages of infection providing a protective role. However, the expression of LL37 is reduced as the infection progresses and results in disturbances in cell turnover in the gastrointestinal tract contributing to *H. pylori*-associated carcinogenesis in the GI tract. Promotion of carcinogenesis and inflammation is attributed to dysregulation of cathelicidin (mechanism unknown). Collectively, these findings suggest that deficiency in this peptide may aid in inflammation and tumor progression (24). So what is the precise role of these AMP during inflammation and carcinogenesis? In addition to its antimicrobial ability, LL37 seems to have a chemotactic capability facilitating the migration of neutrophils, monocytes, CD4^+^ cells, and mast cells. LL37 also has the ability to induce degranulation and release of inflammatory mediators from mast cells, which are one of the first immune cells that encounter invading microbes. It has been suggested that LL37 augments the host’s immune response via regulation of the expression of particular genes with anti-inflammatory and proinflammatory roles (25). In addition, LL37 enhances the induction phase of adaptive immunity through recruitment of T helper cells and increased HLA-DR expression by human dendritic cells (26). Thus, Cathelicidins seem to have a role in augmenting both innate host defenses and adaptive immunity and, where activity is high, may aid in establishing an anti-tumor response.

Defining the exact role that LL37 plays during carcinogenesis is not straight forward. In some studies, expression of LL37 was found to increase in a number of tumors such as ovarian, lung, and breast cancers. On the other hand, the same peptide was found to possess a suppressive role in other types of cancers such as GI tract cancer. This observation was further supported by the finding that Cathelicidin-deficient mice had increased susceptibility to carcinogen-induced colonic tumors. Further studies have shown that LL37 exerts an apoptogenic function via a caspase-independent manner (27). Furthermore, it was shown that lung tumor growth is promoted by cathelicidin expressed in tumor cells (28).

The role that LL37 plays during carcinogenesis might be related to whether the cancer is the result of persistent inflammation or if the inflammation is a result of cancer.

## The Anti-Tumoral Activity of Some AMP and Complement Components Against Hematological Tumors

Hematological tumors such as leukemias and lymphomas cause approximately 10% of deaths in cancer patients. Thus, there is a demand to develop new anti-tumoral agents preferably from natural and biological sources, which targets tumor cells. In addition to their antimicrobial properties, some AMPs seem to exert a cytotoxic effect against tumor cells. Recent studies have focused on exploring the anti-tumoral activity of some AMPs and presenting them either as alternatives to conventional anti-tumor therapies or to be used in combination with other conventional anti-tumor drugs. It is thought that the anti-tumoral activity of these peptides occurs mainly via apoptosis and necrosis. However, the exact mechanism by which some AMPs exert their anti-tumoral activity is not fully understood yet. There have been many speculations in this regard; one of the suggestions is that cell death of tumor cells is due to a detergent-like effect and cell permeabilization. On the other hand, one group has demonstrated that tumor cell death may be a result of calcium accumulation in the mitochondria. It has also been proposed that the anti-tumoral activity of some AMP is a result of the cationic nature of most of these peptides. It is thought that the selective killing of tumor cells is the end result of an interaction between the cationic peptides and the anionic cell membrane components. Certain AMPs can be used with conventional chemotherapeutic drugs to enhance their cytotoxic activity. An example of such AMPs is Cecropin A (CA), which has a cytotoxic effect on human lymphoblastic leukemia and can be used as a pharmacological tool with some chemotherapeutic drugs such as CA/S–FU combination (29).

In addition to AMPs, complement components also seem to have an anti-tumoral action as it had been well documented that leukemic cells have the ability to activate both alternative and lectin pathways of complement. This activation leads to opsonization of tumor cells and their subsequent uptake by NK cells and leukocytes [reviewed in Ref. (30)].

## Antimicrobial Peptides as Novel Anti-Tumoral Therapies

Although a number of studies have demonstrated that some AMPs exhibit anti-tumoral activity against different types of cancers such as leukemia, ovarian, and prostate tumors; however, none of these peptides are currently used commercially to target tumor cells. The idea of using bacteria as anti-tumoral agents was first addressed by two German scientists; W. Busch and F. Fehleisen. These two physicians had individually noted that infections with *Streptococcus pyogenes* in some cancer patients resulted in a regression of tumor cells. However, the American physician William Coley was the first to start a well-documented approach to use bacteria in treatment of cancer back in 1890. Coley had managed to develop a vaccine, which combined two killed bacterial species and used it to treat lymphomas, sarcomas, melanomas, and myelomas with a high success rate. In addition, some anaerobic bacteria have been shown to selectively target solid tumors and leading to tumor lysis. However, this approach was abandoned after the introduction of radiation, chemotherapy, and surgery as standard tools for cancer treatment. Side effects associated with these aforementioned therapies or difficult locations of tumors have stressed the need to look for novel therapies for cancer treatment, which would either replace or supplement conventional cancer therapies. Hence, the use of bacteria and AMPs in cancer treatment has been taken up again. It is thought the cationic nature of the AMPs enable them to interact with the various anionic molecules such as sialic acid residues, heparan sulfate, and anionic phosphatidylserine, which are largely present on the surface of cancer cells. This explains why AMPs are more prone to interact with tumor rather than non-tumor cells. A number of *in vitro* and *in vivo* studies have given evidence on the anti-tumoral activity that some AMPs possess (31). Melittin is an AMP extracted from the European honey bee *Apis mellifera* induces cell lysis. A conjugate of melittin/avidin engineered to target cancer cells with high matrix metalloproteinase 2 (MMP2) activities lead to a significant decrease in the size of B16 tumor in mice (32). A recent study has proposed using BD2 in immunogene therapy of tumors. This strategy involves recruitment of immature dendritic cells to the tumor microenvironment and subsequently promoting their maturation, which will provoke a local anti-tumor immune response (33).

Antimicrobial peptides are not selective against tumor cells. Thus, further systemic investigations into these AMPs are needed to understand the molecular properties, which make these peptides appropriate for clinical use.

## Outlook

In conclusion, a number of studies have addressed the role of AMP during carcinogeneis and cancer progression. But arrived at contradictary conclusions with regard to anti-tumor or tumor promoting activities, some groups have even suggested that certain AMP might provide an unconventional approach for cancer treatment. In addition to complement components, there is good evidence that certain AMP such as defensins and Cathelicidins influence the mobilization from bone marrow and recruitment of cells in the tumor microenvironment (34).

During inflammation, complement components C5a and C3a are generated leading to chemoattraction of immune cells such as T lymphocytes, monocytes, and eosinophils. Subsequently, the interaction of these components with their receptors leads to release of cytokines and reactive oxygen species resulting in tissue damage. Persistent inflammation leads to the establishment of a microenvironment supportive for tumor growth. Tumor-mediated complement activation provides a continuous source of complement activation bioactive products creating an inflammatory environment supporting tumor growth. It was found that activation of C3aR and C5aR lead to an increase in expression of IL-6 mRNA, which is a potent cytokine capable of stimulating angiogenesis and inhibiting apoptosis in tumor cells (35).

Taken together, both complement components and some anti-microbial peptides of the innate immune defense arm have a role in regulating and trafficking of immune cells during tumorigenesis as well as in modulating the adaptive anti-tumor immune response. Their direct role on tumor cells may be more distinct. Therefore, future studies should attempt to study the role of these two effector systems together rather than in isolation to uncover the relative importance of each and possible additive, exploitable, effects during carcinogeneis and cancer progression.

## Conflict of Interest Statement

The authors declare that the research was conducted in the absence of any commercial or financial relationships that could be construed as a potential conflict of interest.
